# Nosocomial transmission in a monkeypox virus clade Ib outbreak, Ireland, August to October 2025

**DOI:** 10.2807/1560-7917.ES.2025.30.50.2500926

**Published:** 2025-12-18

**Authors:** Mark McLoughlin, Laura Fahey, Michael Carr, Billie Caceda, Derval Igoe, Jonathan Dean, Dominic Rowley, Alan Rice, Brian Keogan, Cillian De Gascun, Daniel Hare, Mary Ward

**Affiliations:** 1 Health Service Executive Department of Public Health, Dublin, Ireland; 2 National Virus Reference Laboratory, University College Dublin, Dublin, Ireland; 3 International Collaboration Unit, International Institute for Zoonosis Control, Hokkaido University, Sapporo, Japan; 4 Health Service Executive, Westmeath, Ireland; 5 Health Service Executive National Health Protection Office, Dublin, Ireland; 6 Health Service Executive, Dublin, Ireland

**Keywords:** Ireland, healthcare-associated infections, nosocomial infection, mpox virus (MPXV), clade Ib, infection control, outbreaks, post-exposure prophylaxis, laboratory surveillance

## Abstract

In August–October 2025, an mpox outbreak with monkeypox virus clade Ib was identified in Ireland, involving four epidemiologically linked cases. The cluster originated from a traveller returning from Pakistan via the United Arab Emirates and includes a nosocomial infection. Phylogenetic analysis revealed genetic clustering with an Omani sequence, suggesting Eastern Mediterranean Region transmission routes. This outbreak underscores the importance of clinical vigilance, rapid molecular diagnostics and coordinated public health responses to prevent onward clade Ib transmission in non-endemic countries.

In August 2024, World Health Organization (WHO) declared a Public Health Emergency of International Concern (PHEIC) due to widespread human-to-human monkeypox virus (MPXV) clade I transmission in the Democratic Republic of the Congo (DRC) and neighbouring African countries [1]. We report the first outbreak of mpox due to MPXV clade Ib in Ireland alongside the first reported nosocomial infection outside of the African continent, to the best of our knowledge. This outbreak consisted of one probable mpox case and three epidemiologically linked laboratory-confirmed cases, as defined by the Health Protection Surveillance Centre (HPSC), Ireland [2].

## Case descriptions

Case 1, the primary case, was a male in their thirties with a travel history to Pakistan, returning to Ireland through a connecting flight in the United Arab Emirates in mid-August 2025. This case reported an initial influenza-like illness including pyrexia during transit to Ireland which, on day 3 post-symptom onset, progressed to penile pustular lesions. They attended their general practitioner (GP) on day 4 but a swab was not taken. They did not require further medical assessment and recovered within a week of symptom onset. A swab on day 25 was reported PCR negative for MPXV. No contact with persons with mpox-compatible symptoms or sexual contact before onset was reported. Case 1 lived in a household with Case 2 and 3.

Case 2 was a female in their twenties who had sexual contact with Case 1, 2 days before developing a vulval rash. They attended their GP, but a swab was not taken. By day 9, symptoms had progressed to include pyrexia, sore throat, night sweats and vulval/perineal lesions which later developed to a full-body rash with > 100 lesions. Treatment required in-patient hospital admission where tecovirimat was initiated and continued for 14 days. Their mpox diagnosis was confirmed as MPXV clade Ib by pan-orthopoxvirus [3] and MPXV clade [4] and subclade-specific [5] real-time quantitative PCR (qPCR) assays. Case 2 was discharged after 11 inpatient days to complete recovery at home.

Case 3, a young child, was a household contact of Case 1 and 2. The clinical presentation of Case 3 included pyrexia, coryza and widespread vesicular rash which required clinical assessment and antiviral treatment without inpatient admission. The mpox diagnosis was confirmed as MPXV clade Ib by qPCR.

Case 4 was a female healthcare worker (HCW) in their twenties, who provided clinical care for Case 2 during their inpatient admission before the inclusion of mpox as a differential diagnosis. No high-risk activities were reported, and Case 4 applied the recommended contact and droplet precautions of personal protective equipment (PPE) (apron, gloves, surgical mask). Case 4 reported a rash on their arm which developed into five lesions on their hand, elbow, leg and back. This case did not require hospital admission. Their mpox diagnosis was confirmed as MPXV clade Ib by qPCR.

**Figure 1:**
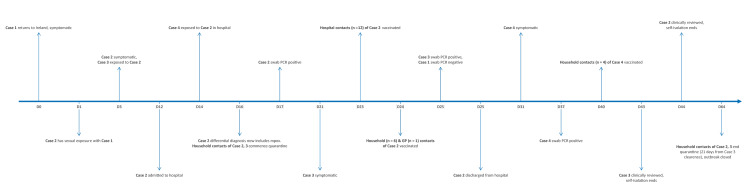
Timeline of disease onset in cases in an mpox outbreak of monkeypox virus clade Ib, Ireland, August–October 2025 (n = 4)

The timeline of each case is shown in Figure 1.

GP: general practitioner.

## Laboratory diagnostics and sequencing

In August–September 2025, MPXV qPCR testing detected pan-orthopoxvirus and MPXV clade Ib DNA from swabs taken from Case 2, 3 and 4 (Table). Testing for herpes simplex virus and varicella-zoster virus DNA was performed on all three individuals, and neither were detected.

**Table:** Detection and clade assignment of monkeypox virus from three cases in an mpox outbreak, Ireland, August–October 2025^a^

Case	Sample type	Cq value				Genome coverage (%)
		Pan-orthopoxvirus	MPXV clade Ia	MPXV clade Ib	MPXV clade II	
Case 2	Swab (vulval)	23.06	Not detected	20.85	Not detected	98.11
Case 3	Swab (nasal and skin)	31.1	Not detected	27.68	Not detected	86.54
Case 4	Swab (skin)	22.01	Not detected	18.89	Not detected	97.79

Cq: quantification cycle; MPXV: monkeypox virus.

^a^ Of note, Case 1 was swabbed on day 25 and was negative for MPXV. Earlier swabbing for Case 1 was not possible.

Cq values obtained via quantitative real-time PCR on samples taken from the three cases; the first laboratory-confirmed case (Case 2), the household contact (Case 3) and the healthcare worker (Case 4). Genome coverage percentages are reported relative to the masked reference genome (KJ642613.1_masked).

Whole genome sequencing (WGS) analysis [6-8] confirmed the MPXV clade Ib sequences from Case 2, 3 and 4 were genetically identical over the regions sequenced. This confirmed the epidemiological links and excluded MPXV infection from other clades or independent clade Ib infections from another source.

**Figure 2:**
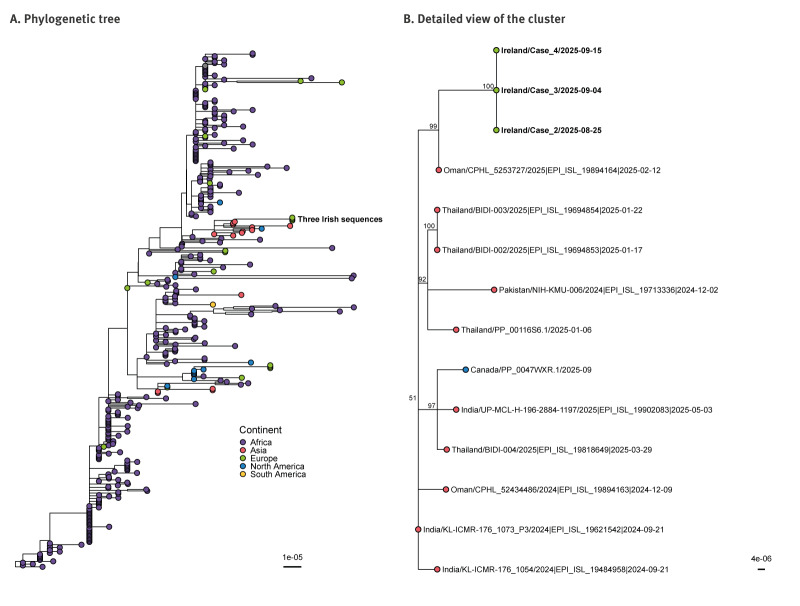
Phylogenetic tree of monkeypox virus clade Ib sequences of cases in Ireland (n = 3), August–October 2025 and global sequences (n = 370), October 2023–September 2025

Phylogenetic analysis was performed using 370 MPXV genome sequences from the Pathoplexus (https://pathoplexus.org/) and GISAID (https://gisaid.org/) databases, with duplicate sequences removed, as presented in Supplement. Multiple sequence alignment was performed using SQUIRREL [9] (version 1.0.12; https://github.com/aineniamh/squirrel), and a maximum likelihood phylogeny was inferred with IQ-TREE [10] (version 3.0.1). The three MPXV clade Ib genome sequences were genetically indistinguishable and clustered together on the phylogenetic tree with full bootstrap support (100; Figure 2).

MPXV: monkeypox virus.

Panel A. The full phylogenetic tree is visualised.

Panel B. An expanded view of the cluster containing the three Irish MPXV genome sequences and closely related international sequences. Sequence labels include the country and date of collection. Branch support values shown are based on ultrafast bootstrap replicates (n = 10,000).

Maximum-likelihood phylogeny of 373 MPXV clade Ib virus genomes, including the three Irish genome sequences, generated using IQ-TREE (substitution model: HKY). Tip points are coloured by the continent of sample collection, as indicated in the legend. The scale bar represents the number of nt substitutions per site.

The shortest genetic distance on the phylogeny identified was to a sequence collected in Oman from February 2025 (GISAID ID: EPI_ISL_19894164). The resulting cluster of four sequences was strongly supported (bootstrap = 99). Six observed mutations were unique to the three Irish MPXV genomes relative to all other sequences in the tree, five of which occurred within GA or TC dinucleotide contexts consistent with APOBEC3-mediated editing (G2204A, G59787A, C62338T, G74989A, C90799T) and one within a dinucleotide GT context (G151305A). This mutational signature is consistent with human-to-human as opposed to zoonotic transmission [11]. Two additional mutations (G40646A and G188953A) were shared between the Irish and Omani (EPI_ISL_19894164) sequences and are also putatively the result of APOBEC3 editing. A broader clade (Figure 2) was identified, comprising the four sequences above and 11 additional sequences, predominantly from Asia, with one from Canada. This is noteworthy given that only 15/370 (4%) of clade Ib sequences in the full phylogenetic dataset originated from Asia, while most (n = 327; 88%) were from Africa.

## Public health measures

Public health (PH) services, responsible for epidemiological investigation of this outbreak, were notified on the day of diagnosis of Case 2. They performed contact tracing for Case 2, 3 and 4 per national guidance [12], including community and healthcare settings. Based on risk assessment, the PH services provided relevant information on symptoms and appropriate isolation to contacts. This included tailored information leaflets, warn and inform letters for hospital staff and communications to relevant schools [13].

The national guidelines also advocated for post-exposure vaccination in persons with a high or intermediate exposure [14]. Contacts within the community were identified and risk assessed for vaccination by PH services, before receiving vaccination from mobile vaccination services. Hospital contacts (n = 12) received vaccination from clinical services within the hospital. In addition, all household contacts (n = 10) and all GP contact (n = 1) were vaccinated (Figure 1).

Once mpox was included as a differential diagnosis (Figure 1), infection prevention and control (IPC) measures for Case 2 included isolation in a negative-pressure room under airborne/contact/droplet precautions. All personnel used full PPE (FFP3 respirators, gowns, gloves and eye protection) and enhanced cleaning with chlorine-based disinfectants was undertaken. Enhanced environmental decontamination and waste management protocols were applied, in line with WHO [15] and national guidance [16]. Laboratory handling was minimised, suspected clinical samples were referred directly to the National Virus Reference Laboratory (NVRL), Dublin, Ireland, for MPXV testing.

## Discussion

This report represents, to the best of our knowledge, the first reported nosocomial transmission of MPXV clade Ib outside of Africa, occurring alongside the ongoing rise of MPXV clade Ib importations in countries outside Middle Africa [17] as highlighted by European Centre for Disease Prevention and Control (ECDC) [18] and WHO [19]. This cluster highlights the ongoing possibility of mpox transmission from travel-associated cases and emphasises the need for a high level of clinical suspicion of mpox in cases with relevant clinical symptoms associated with travel to countries where mpox is currently circulating.

While the source of infection of Case 1 cannot be ascertained from their history, phylogenetic findings were informative in identifying the potential origin of infection. The three Irish MPXV clade Ib genomes form a strongly supported cluster with an international genome from Oman (EPI_ISL_19894164), which is relevant given Case 1’s recent travel history to the Eastern Mediterranean Region. However, currently there is a paucity of genomic data which prevents laboratory confirmation of transmission from this area. Case 2 is the most likely source of transmission to Case 3, before their hospital admission, given their frequent close interaction in their shared home setting. Similarly, Case 2 is the most likely source of contact transmission to Case 4, whose clinical presentation was consistent with local inoculation of exposed skin on their arm.

In this outbreak, clinical diagnosis of mpox was a challenge in both primary and secondary healthcare settings. Early recognition of mpox in Case 2’s hospital presentation was complicated by their absence of travel history and initial clinical presentation that did not include expected skin lesion counts or respiratory symptoms [20]. As a result, implementation of contact and airborne transmission-based IPC precautions including full sleeve gowns and respirators was delayed until mpox was clinically included in the differential. This event highlights possible diagnostic difficulty, particularly in patients presenting with vesiculopustular lesions and systemic symptoms and emphasises the importance of clinical consideration of mpox outside of links to endemic settings in both primary and secondary healthcare settings.

Additional challenges faced by PH colleagues included the identification of contacts and initial vaccination hesitancy. These challenges were managed through liaising with community healthcare services, working with key primary healthcare professions and seeking expert paediatric consultation on case management and vaccine administration. Access to these key personnel and services on an ongoing 24/7 basis is essential to the ability to implement appropriate public health control measures, as per national [12] and international [21] recommendations.

Despite these challenges, the management of this cluster highlights the strength of multiple healthcare stakeholders working together to respond to an outbreak of an emerging infectious disease. This collaborative effort was key to limiting the spread of MPXV clade Ib while gaining insight into the most likely routes of transmission. Similar scenarios and challenges are likely to be experienced by other European countries, requiring clinical vigilance, access to clade-specific identification/genomics and a robust public health response service.

## Conclusion

This report highlights the ongoing threat that MPXV clade Ib poses, echoed recently by both ECDC and WHO. Multi-organisational responses, including prompt case identification in both primary and secondary healthcare settings, timely molecular diagnostics and a robust public health system, are imperative to prevent onward transmission events. The findings are relevant in the context of undetected transmission of mpox due to MPXV clade Ib internationally and the rise of autochthonous cases in the European Union/European Economic Area (EU/EEA) countries without an associated travel history.

## Data Availability

The consensus genome sequence generated for Case 2 in this study was made publicly available on Pathoplexus (https://pathoplexus.org/; accession ID: PP_003V57M.1). The corresponding raw sequencing data are available in the Sequence Read Archive (SRA; https://www.ncbi.nlm.nih.gov/sra) under project accession number PRJNA1366958.

## Supplementary Data

102000 Supplementary Material
