# Supplementary material for: Nosocomial transmission in a monkeypox virus clade Ib outbreak, Ireland, August to October 2025
Source: Euro Surveill. 2025 Dec 18;30(50):2500926. doi: 10.2807/1560-7917.ES.2025.30.50.2500926 (PMC12719937; doi:10.2807/1560-7917.ES.2025.30.50.2500926)
Supplement: Supplementary Material [file 25-00926_MCLOUGHLIN_Supplement.pdf]

Supplement S1: Genome sequences used in this study

This supplementary material is hosted by Eurosurveillance as supporting information alongside the article “Nosocomial transmission in a monkeypox virus clade Ib outbreak, Ireland, August to October 2025”, on behalf of the authors, who remain responsible for the accuracy and appropriateness of the content. The same standards for ethics, copyright, attributions and permissions as for the article apply. Supplements are not edited by Eurosurveillance and the journal is not responsible for the maintenance of any links or email addresses provided therein.

Supplementary Table 1: GISAID and Pathoplexus genome sequences used in this study.

Identifier	Digital Object Identifier	Number of individual viruses	Data Collection range	Number of countries/territories
EPI_SET_251218bo	https://doi.org/10.55876/gis8.251218bo	235	2023-11-02 to 2025-05-03	14
PP_SS_430.1	https://doi.org/10.62599/PP_SS_430.1	135	2023-10-05 to 2025-09	10

All GISAID genome sequences and associated metadata supporting the findings of this study can be accessed through the persistent digital object identifier **https://doi.org/10.55876/gis8.251218bo**. In addition to the minted DOI, GISAID also communicates the aggregation of GISAID accession numbers (EPI_ISL_IDs) through the corresponding EPI_SET_251217at identifier to facilitate both, the acknowledgment of all data contributors and the direct retrieval of the underlying data from GISAID used in this study.
